# Adherence to the Western, Prudent and Mediterranean Dietary Patterns and Colorectal Cancer Risk: Findings from the Spanish Cohort of the European Prospective Investigation into Cancer and Nutrition (EPIC-Spain)

**DOI:** 10.3390/nu14153085

**Published:** 2022-07-27

**Authors:** Adela Castelló, Miguel Rodríguez-Barranco, Nerea Fernández de Larrea, Paula Jakszyn, Ane Dorronsoro, Pilar Amiano, María-Dolores Chirlaque, Sandra Colorado-Yohar, Marcela Guevara, Conchi Moreno-Iribas, Marina Pollán, María-José Sánchez

**Affiliations:** 1 Cancer and Environmental Epidemiology Unit, National Centre for Epidemiology, Instituto de Salud Carlos III, 28029 Madrid, Spain; acastello@isciii.es (A.C.); nfernandez@isciii.es (N.F.d.L.); mpollan@isciii.es (M.P.); 2 Consortium for Biomedical Research in Epidemiology and Public Health (CIBERESP), 28029 Madrid, Spain; epicss-san@euskadi.eus (P.A.); mdolores.chirlaque@carm.es (M.-D.C.); sandram.colorado@carm.es (S.C.-Y.); mp.guevara.eslava@navarra.es (M.G.); mc.moreno.iribas@navarra.es (C.M.-I.); mariajose.sanchez.easp@juntadeandalucia.es (M.-J.S.); 3 School of Medicine, University of Alcalá, 28871 Alcalá de Henares, Spain; 4 Andalusian School of Public Health, 18011 Granada, Spain; 5 Instituto de Investigación Biosanitaria ibs. GRANADA, 18012 Granada, Spain; 6 Unit of Nutrition and Cancer, Cancer Epidemiology Research Program, Catalan Institute of Oncology-IDIBELL, L’Hospitalet de Llobregat, 08908 Barcelona, Spain; paujak.ico@gmail.com; 7 Facultat de Ciències de la Salut Blanquerna, Universitat Ramon LLul, 08021 Barcelona, Spain; 8 Sub-Directorate for Public Health and Addictions of Gipuzkoa, Ministry of Health of the Basque Government, 20013 San Sebastian, Spain; a-dorronsoroerauskin@euskadi.eus; 9 Epidemiology of Chronic and Communicable Diseases Group, Biodonostia Health Research Institute, 20014 San Sebastian, Spain; 10 Department of Epidemiology, Regional Health Council, IMIB-Arrixaca, 30008 Murcia, Spain; 11 Social-Health Department, Murcia University, 30008 Murcia, Spain; 12 Research Group on Demography and Health, National Faculty of Public Health, University of Antioquia, Medellín 050010, Colombia; 13 Navarra Public Health Institute, 31003 Pamplona, Spain; 14 Navarra Institute for Health Research (IdiSNA), 31008 Pamplona, Spain; 15 Department of Preventive Medicine and Public Health, University of Granada, 18071 Granada, Spain

**Keywords:** dietary patterns, Western diet, Mediterranean diet, colorectal neoplasms

## Abstract

The aim of this study was to explore the association between three previously identified dietary patterns (Western, Prudent, and Mediterranean) and colorectal cancer (CRC) risk by sex and cancer subtype. The Spanish cohort of the European Prospective Investigation into Cancer and Nutrition study provided dietary and epidemiological information from 15,629 men and 25,808 women recruited between 1992 and 1996. Among them, 568 CRC cases and 3289 deaths were identified during a median follow-up of 16.98 years. The associations between adherence to the three dietary patterns and CRC risk (overall, by sex, and by tumour location: proximal and distal colon and rectum) were investigated by fitting multivariate Cox proportional hazards regression models stratified by study centre and age. Possible heterogeneity of the effects by sex and follow-up time (1–10 vs. ≥10 years) was also explored. While no clear effect of the Prudent dietary pattern on CRC risk was found, a suggestive detrimental effect of the Western dietary pattern was observed, especially during the first 10 years of follow-up (HR_1SD-increase_ (95% CI): 1.17 (0.99–1.37)), among females (HR_1SD-increase_ (95% CI): 1.31 (1.06–1.61)), and for rectal cancer (HR_1SD-increase_ (95% CI): 1.38 (1.03–1.84)). In addition, high adherence to the Mediterranean pattern seemed to protect against CRC, especially when restricting the analyses to the first 10 years of follow-up (HR_1SD-increase_ (95% CI): 0.84 (0.73–0.98)), among males (HR_1SD-increase_ (95% CI): 0.80 (0.65–0.98)), and specifically against distal colon cancer (HR_1SD-increase_ (95% CI): 0.81 (0.63–1.03)). In conclusion, low adherence to the Western diet and high adherence to the Mediterranean dietary pattern could prevent CRC, especially distal colon and rectal cancer.

## 1. Introduction

The latest global cancer statistics indicate that in 2020 colorectal cancer (CRC) was the third-most frequently diagnosed tumour and the second cause of cancer deaths globally [1]. This tumour has been associated with physical inactivity, obesity, high intake of red and processed meat and alcohol, low intake of fibre, and smoking, among other less relevant factors [2]. According to the latest Word Cancer Research Fund and American Institute for Cancer Research (WCRF/AICR) report on diet, nutrition, physical activity, and CRC from 2018, there is strong evidence for a protective effect of whole grains, dietary fibre, dairy products, and calcium intake and a detrimental effect of processed and red meat and alcohol consumption on CRC incidence, while the data for other foods, nutrients, and dietary patterns is still considered insufficient to draw firm conclusions [3]. 

One possible explanation for this lack of conclusive evidence may be that most studies focus on exploring the effect of individual foods and nutrients on the risk of CRC [2,4], even though foods and nutrients are not consumed individually. Dietary patterns take into account the interactions between individual dietary factors [5], and their use overcomes the limitations of studying individual foods or nutrients. Two main types of diet quality indices are used to investigate the association between dietary patterns and risk of disease: “a priori” (investigation driven) and “a posteriori” (data driven) dietary patterns. The latter have the advantage of being extracted with statistical methods using the dietary information of the sample under study, which ensures their independence from the disease and their representativeness of the diet of the individuals in the study. 

Multiple studies have explored the association between “a posteriori” dietary patterns and CRC risk [6,7,8,9,10,11,12,13,14,15,16,17,18,19,20,21,22,23,24,25,26]. All of them agree in the identification of a healthy-type dietary pattern, usually labelled as Mediterranean/Healthy/Prudent, which, in most cases, appears as protective against total CRC [10,13,14,15,17,18,19,20,21,22,24,25,26] and/or specifically against colon [6,8,10,12,13,14,21,22,26] or rectal cancer [10,13,18,19,21,22,26]. They also identify a Western-type dietary pattern, frequently associated with an increased risk of total colorectal [11,13,14,15,17,19,20,21,22,23,25,26], colon [6,8,9,10,13,14,16,19,21,22,26], and/or rectal [13,14,16,18,19,21,22,26] cancer.

In a previous study conducted in Spain, three dietary patterns (Western, Prudent, and Mediterranean) showed some associations with breast cancer risk [27]. After testing the applicability [28] of these patterns in different settings, they were applied in another case–control study that showed associations with other tumours in both males and females [27,29,30,31], including CRC [26]. 

The aim of the current study was to apply these three dietary patterns [27] to data from the European Prospective Investigation into Cancer and Nutrition Spanish cohort (EPIC-Spain) to validate the previous findings [26] and explore the association of the dietary patterns with CRC by sex and tumour location.

## 2. Materials and Methods

### 2.1. Study Population

The European Prospective Investigation into Cancer and Nutrition (EPIC) is a multicentre cohort study designed to investigate the relationship between lifestyle, diet, environmental factors, and cancer [32,33,34]. For the present work, data from the Spanish cohort (EPIC-Spain) were selected. The EPIC-Spain cohort recruited, between 1992 and 1996, 41,437 healthy adults (25,808 women and 15,629 men) aged 29–69 from the general population in five northern (Asturias, Gipuzkoa, and Navarra) and southern (Murcia and Granada) Spanish provinces, including the Mediterranean shore. Further details about the EPIC cohort can be found elsewhere [32,33,34].

Data on the sociodemographic characteristics, physical activity, smoking, alcohol consumption, and medical history of previous illnesses were collected in a personal interview. At the same appointment, anthropometric measurements (height, weight, and waist circumference) were taken by trained personnel using standardised procedures. The usual diet throughout the year before recruitment, accounting for seasonal variations, was obtained with a computerised dietary history questionnaire previously validated in Spain and administered by trained interviewers [35,36]. 

The ethical review boards from the International Agency for Research on Cancer (IARC) (Lyon, France) and the Medical Ethics Committee of Bellvitge Hospital (L’Hospitalet de Llobregat, Spain) approved the study, and all the participants gave their written informed consent.

### 2.2. Cases Ascertainment and Follow-Up Period

Cases were defined as first occurrence of a primary malignant tumour of the colon (C18 of the ICD-10) or rectum (C19–C20). Tumours originating in the proximal area of the splenic flexure (cecum, ascending colon, and transverse colon) were categorised as proximal colon cancers (C18.0–C18.5), those originating in the descending (C18.6) or sigmoid colon (C18.7) were classified as distal colon cancers, and those located at the recto sigmoid junction (C19) or rectum (C20) were grouped as rectal cancers. Tumours with overlapping lesion of colon (C18.8) or those with non-specified locations (C18.9) were excluded from the analyses by subtype. Cancer cases were identified by linking the data from the population-based cancer registries of the five mentioned regions with the EPIC-Spain information. Dates and causes of death were extracted from the population-based mortality registry of the National Institute of Statistics. 

The first-year follow-up was excluded from the analyses to avoid reverse causation due to silent tumours not diagnosed at the time of recruitment that might have affected the diets of participants during the months prior to the interview. Therefore, the follow-up period was defined as the time from one year after the date of recruitment to the CRC diagnosis, diagnosis of other tumours, and death or last completed follow-up date, depending on which occurred first. The censoring dates for the last complete follow-up were 31 December 2010 for Asturias, 31 December 2011 for Navarra, 31 December 2012 for Granada, 30 December 2013 for Gipuzkoa, and 31 December 2013 for Murcia.

### 2.3. Adherence to Dietary Patterns

Three previously identified dietary patters [27] were analysed: (a) the Western dietary pattern, characterised by high intakes of high-fat dairy products, processed meat, refined grains, sweets, caloric drinks, convenience food and sauces, and low intakes of low-fat dairy products and whole grains; (b) the Prudent dietary pattern, which represents high intakes of low-fat dairy products, vegetables, fruits, whole grains and juices; and (c) the Mediterranean dietary pattern, which consists of high intakes of fish, vegetables, legumes, boiled potatoes, fruits, olives, and vegetable oil and a low intake of juices. 

These patterns were originally identified in the sample of controls of the EpiGEICAM case–control study [27] by applying the principal components analysis (PCA) on 26 food groups. This method provides a set of weights (pattern loadings) associated with each food group that represents a correlation between the food intake and pattern scores and can be used to reproduce such patterns in other samples, as explained in detail elsewhere [28]. Briefly, the food intake information collected with the EPIC-Spain dietary history questionnaire (excluding noncaloric and alcoholic beverages) was classified into these 26 food groups. Since the food supply in 1992 (EPIC) was somewhat different from the food supply in 2006 (EpiGEICAM), the methodology for grouping some foods such as dairy and fish was different in these two studies. To obtain comparable food groups, a set of weights was defined to distribute these food groups using the data from the 1998 food consumption panel elaborated by the Spanish Ministry of Agriculture, Food and Environment (MAPAMA) [37]. The distribution of the intake of these food groups among cancer and noncancer cases observed in the multi-case–control study of cancer MCC-Spain [38] was also taken into account (Table S1). 

Finally, adherence scores for the Western, Prudent, and Mediterranean dietary patterns were calculated as a linear combination of the pattern loadings for each food group and patterns obtained in the EpiGEICAM study [27] and the food group consumption reported by the participants of the EPIC-Spain study. The adherence to these three dietary patterns was modelled as a categorical variable (quartiles of the distribution for the whole EPIC sample) and as a continuous variable (original score and one standard deviation increment in the score). 

### 2.4. Statistical Analyses

The distribution of quantitative variables according to the quartiles of adherence to the Western, Prudent, and Mediterranean dietary patterns was described with the median and interquartile range (IQR), and the significance of the differences were assessed with Kruskal–Wallis tests. For the qualitative variables, a number of cases and percentages were used for the description and chi-squared tests (ignoring missing values) for hypothesis testing. 

Crude and adjusted associations between CRC incidence and the adherence to the Western, Prudent, and Mediterranean dietary patterns were assessed by fitting Multivariate Cox proportional hazards regression models stratified by centre and age (5-year groups) and adjusted for sex, BMI, physical activity, lifetime alcohol intake, smoking habit, total energy intake (including all foods and beverages), education, and family history of CRC. In the case of the Western dietary pattern, the models were also adjusted for adherence to the Prudent and Mediterranean dietary patterns. In the case of the Prudent and Mediterranean dietary patterns, the models were also adjusted for adherence to the Western dietary pattern. A possible heterogeneity of the effects by sex was explored by including in the models an interaction between sex and adherence in each of the three patterns (three different models with one interaction in each one). Analogous analyses were performed considering the tumour location (proximal colon, distal colon, and rectum). Finally, nonlinear associations were modelled through restricted cubic splines with knots at the 5th, 35th, 65th, and 95th percentiles, as recommended by Harrell [39].

**Figure 1:**
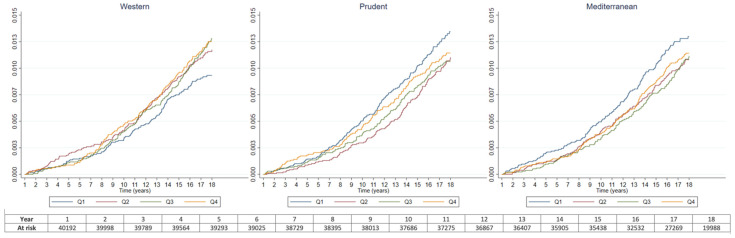
Adjusted ^a^ cumulative incidence of colorectal cancer by quartiles of adherence to the Western, Prudent, and Mediterranean dietary patterns. ^a^ Adjusted for lifetime alcohol intake, smoking habit, energy intake, BMI, physical activity, education, sex, family history of colorectal cancer, age at recruitment, and centre. For the Western dietary pattern, also adjusted for adherence to the Prudent and Mediterranean dietary patterns. For the Prudent and Mediterranean dietary patterns, also adjusted for adherence to the Western dietary pattern.

Compliance with the proportional hazards assumption was checked visually with graphs of the adjusted Kaplan–Meier failure estimates for CRC by quartiles of adherence to each pattern (Figure 1, Figures S1 and S2) and numerically by testing the nonzero slope in a generalised linear regression of the scaled Schoenfeld residuals on time. The violation of the assumption of proportional hazards for the age estimates in most of these models was fixed by stratifying the models by age. In addition, the proportional hazards assumption was violated for some sex estimates in the analyses by tumour location, which was also resolved by stratifying them by sex. 

A sensitivity analysis was performed by obtaining the HR for the five and ten first years of follow-up to explore possible dilution of the effects over the years caused by the loss of representativeness of the dietary information collected at recruitment. Risks were obtained by splitting the database into two follow-up periods (1–5 vs. >5 years and 1–10 vs. >10 years) and the estimates were obtained by including in the models an interaction term between the periods and the level of adherence to the three dietary patterns explored. Since the results obtained for both analyses were similar, we show the estimations for the second classification (≤1–10 vs. >10 years follow-up).

All analyses were performed using Stata/MP version 16 (Statacorp, College Station, TX, USA), and a fixed 95% confidence level was stablished for all analyses except for the interaction analyses for which a 90% confidence level was set.

## 3. Results

After excluding 351 participants (342 non-cases and 9 cases) due to implausible energy intakes below 750 or above 4500 kcals per day, 6 (all non-cases) with a BMI over 60 and the first year of follow-up (165 non-cases and 17 cases), the final sample size included 40,898 individuals (15,368 males and 25,530 females). Among them, 568 CRC cases and 3289 deaths were identified during a median follow-up of 16.98 years (10.54 years for cases and 11.13 years for deaths). 

Participants in the upper quartiles of adherence to the Western dietary pattern showed a markedly higher alcohol and energy intake, slightly lower physical activity and education level, and smoked more. In the lower quartiles of adherence to this pattern, more females and younger participants with lower BMIs were found. As for the Prudent dietary pattern, the highest alcohol intake was observed for the second quartile of adherence and the lowest for the first. The energy intake increased and age and BMI slightly decreased with increasing the adherence to this pattern. Although statistically significant, no major differences were found by sex and family history of CRC, and participants with a higher adherence to the Prudent pattern appeared to be slightly more inactive, more educated, and smoked less. Finally, participants with a higher adherence to the Mediterranean dietary pattern showed a significantly higher alcohol and energy intake, were younger, and presented slightly lower BMI. The percentage of females was higher in the lower categories of adherence to this pattern, and higher adherence was associated with lower physical activity, smoking habit, and higher educational level (Table 1).

**Figure 2:**
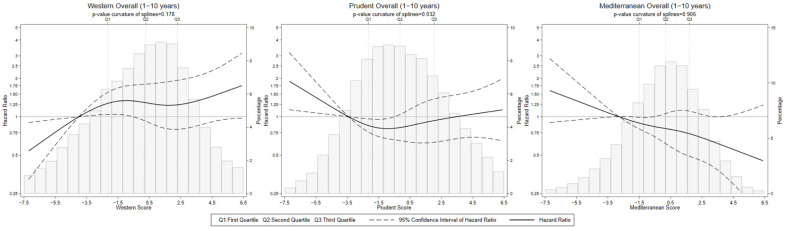
Nonlinear association between colorectal cancer incidence and adherence scores to the Western, Prudent, and Mediterranean dietary patterns for the follow-up period 1–10 years. Full sample. HR of colorectal cancer stratified by centre and age in 5-year periods and adjusted by sex, lifetime alcohol intake, smoking habit, energy intake, BMI, physical activity, education, and family history of colorectal cancer. For Western dietary pattern, also adjusted by the adherence to the Prudent and Mediterranean dietary patterns. For Prudent and Mediterranean dietary patterns, also adjusted by the adherence to the Western dietary pattern.

The results for the full sample and follow-up period (Figure 1 and Figure 2 and Table 2) revealed a positive trend in the association between the Western dietary pattern and CRC risk that gained strength when the analyses were restricted to the first 10 years of follow-up, with a 53% increase in CRC risk for participants in the highest quartile of adherence (HR_Q4vs.Q1_ (95% CI): 1.53 (0.99–2.36)) and a non-negligible positive trend that, despite not being totally linear (Figure 2), seemed to be fairly steady (HR_1SD-increase_ (95% CI): 1.17 (0.99–1.37); *p* for trend = 0.087; *p*-value for the curvature of splines = 0.173). A suggestive protective effect, very similar for the second to fourth quartiles and with a marked linear component (*p*-value for the curvature of splines = 0.906), was also observed for the Mediterranean dietary pattern (HR_1SD-increase_ (95% CI): 0.91 (0.82–1.01)), especially when the last years of follow-up were excluded (HR_1SD-increase_ (95% CI): 0.84 (0.73–0.98)).

**Figure 3:**
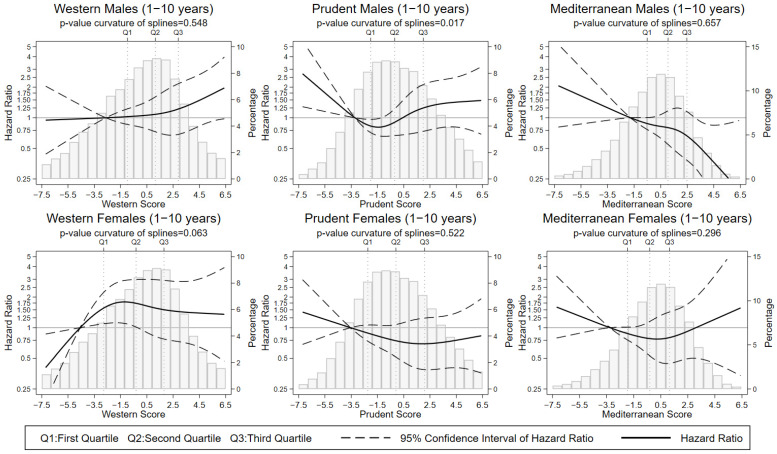
Nonlinear association between colorectal cancer incidence and adherence scores to the Western, Prudent, and Mediterranean dietary patterns for the follow-up period 1–10 years. By sex. HR of colorectal cancer among males and females and stratified by centre and age in 5-year periods and adjusted by lifetime alcohol intake, smoking habit, energy intake, BMI, physical activity, education, and family history of colorectal cancer. For Western dietary pattern, also adjusted by the adherence to the Prudent and Mediterranean dietary patterns. For Prudent and Mediterranean dietary patterns, also adjusted by the adherence to the Western dietary pattern.

When stratifying by sex (Figure 3 and Table 3), we observed that the detrimental effects of high adherence to the Western dietary pattern were only observed among females (HR_Q4vs.Q1_ (95% CI): 1.55 (1.01–2.38)) and for adherence as low as the second quartile, especially when restricting the analyses to the first 10 years follow-up (HR_Q2vs.Q1_ (95% CI): 1.98 (1.16–3.36); HR_Q3vs.Q1_ (95% CI): 1.78 (0.99–3.20); HR_Q4vs.Q1_ (95% CI): 2.16 (1.16–4.02), *p* for interaction with sex = 0.005). This similarity in the associations found for all quartiles might explain the curvature observed in the association between the Western diet and CRC in this subgroup (*p*-value for the curvature of splines = 0.063). In contrast, the protective effect of the Mediterranean diet was specifically observed among males and presented a strong negative trend with a fairly linear shape (*p*-value for the curvature of splines = 0.657) and particularly when excluding the last follow-up years (HR_1SD-increase_ (95% CI): 0.80 (0.65–0.98); *p* for interaction with sex = 0.006).

Finally, an exploration of the associations by tumour location (Table 4) showed that the positive trend found in the association of the Western dietary pattern with CRC was observed specifically for rectal tumours ((HR_Q4vs.Q1_ (95% CI): 2.41 (1.10–5.30) and HR_1SD-increase_ (95% CI): 1.38 (1.03–1.84)) for the first 10 years of follow-up. Similarly, the protective effect of the Mediterranean dietary pattern was only observed for distal colon cancer during the whole period (HR_1SD-increase_ (95% CI): 0.86 (0.73–1.02)) and for the first 10 years of follow-up (HR_1SD-increase_ (95% CI): 0.81 (0.63–1.03)). Nonlinear associations were not reported in this case, because the sample size was too small to obtain precise estimations, but the shape of the splines pointed in the same direction (Supplementary Materials Figure S3). 

No clear associations were found between adherence to the Prudent dietary pattern and CRC risk.

## 4. Discussion

Our results indicate that, while there is no clear effect of the Prudent dietary pattern on CRC risk, moderate to high adherence to the Western dietary pattern might increase the CRC risk, especially among females and mainly for rectal cancer. In addition, a potential protective effect of a high adherence to the Mediterranean dietary pattern was observed, especially for males and against distal colon cancer. Most of the associations gained consistency and strength when restricting the analyses to the first 10 years of follow-up. 

Regarding the validation of the above results, our main findings are mostly in agreement with those of the MCC-Spain study, which showed a strong detrimental effect of the Western dietary pattern and a protective effect of the Mediterranean dietary pattern on CRC risk [26]. However, while no sex differences were found in the MCC-Spain study, in our study, the detrimental effect of the Western dietary pattern was only observed among females, whereas the beneficial effect of the Mediterranean dietary pattern was only observed among males. Furthermore, similar to the current results, a stronger detrimental effect of the Western dietary pattern on distal colon and rectal cancer was found in the MCC-Spain study, although, in the present study, the greater effect on distal colon cancer did not reach significance. The protective effect of the Mediterranean dietary pattern was observed for all sites in MCC-Spain, whereas, in our data, this effect was only found for distal colon cancer. Finally, high adherence to the Prudent dietary pattern was not associated with CRC risk in either study. 

The results of other studies exploring the association between “a posteriori” dietary patterns and CRC risk support the associations found in the present study. The identification of a Western-type dietary pattern is common [6,7,8,9,10,11,12,13,14,15,16,17,18,19,20,21,22,24,25], and most of the studies exploring its association with total CRC [7,10,11,12,13,14,15,17,18,19,20,21,22,24,25] found a detrimental effect [10,11,13,14,15,17,19,20,21,22,25]. The identification of a Mediterranean-type [6,7,8,9,10,11,13,14,15,17,18,20,21,22,23,25] and, less frequently, of a Prudent-type [10,12,13,16,19,21,23,24] diet is also recurrent. While the association of the Prudent pattern with total CRC is controversial, with some studies finding a significant protective [10,13,19,21,24] effect and others not [12,16,23], the protective effect of the Mediterranean diet seems to be more consistent [13,14,15,17,18,20,21,22,25]. Considering the composition of the Western-type dietary pattern (high intake of high-fat dairy products, processed meat, refined grains, sweets, caloric drinks, convenience food and sauces, and low intakes of low-fat dairy products and whole grains), some biological mechanisms support the associations found. On one hand, the intake of fat, red, and processed meats; refined grains; and sweets has been associated with higher levels of inflammatory markers [40] and with inflammation-related chronic diseases [41]. In addition, red meat products are rich in heme iron, which is associated with the generation of free radicals that attack DNA and damage tissues [42,43]. Furthermore, processing or cooking meat at high temperatures can generate carcinogenic substances such as N-Nitroso and polycyclic aromatic hydrocarbons [43,44]. On the other hand, fruits, vegetables, and legumes present in the Mediterranean pattern are rich in antioxidants that may reduce cancer risk by quenching free radicals and reducing oxidative damage to DNA [45]. Additionally, the higher fibre intake among those with a high adherence to this pattern might translate into a healthier gastrointestinal tract, as it helps to dilute the faecal content, decreases the transit time, and increases stool weight [46]. Some studies suggest that olive oil, the main source of fat in the Mediterranean dietary pattern in Spain [37], may induce apoptosis and downregulate the expression of cyclooxygenase2 and Bcl-2 proteins with a crucial role in colorectal carcinogenesis [47]. Finally, the microbiome composition, strongly influenced by dietary habits [48], might also play an important role in colorectal carcinogenesis [49]. A recent review suggests that a diet rich in plants and fibre and low in meat and fat might contribute to a healthier microbiome [50].

Among the studies that found an association of the Western dietary pattern with total CRC, some also explored sex differences [13,14,15,21,22], and the association was frequently observed only in females [14,15,21] or was stronger among females than males [13]. The only study [22] that found no differences performed analyses on two different samples of women (Nurses Health study) and men (Health Professionals Follow-up Study) [22]. The study by de Stefani et al. [14] found two different patterns labelled as Western (fried red meat, barbecue, and eggs) and Traditional (grains, all tubers, desserts, and dairy foods) that characterised the diet of the Uruguayan population. Both were associated with an increased CRC risk but the first one only among males and the second one only among females. These differences in the profiles of men and women highly adherent to the Western dietary pattern could be behind the stronger effect of this pattern in women. In our sample, females reported higher intakes of dairy products, sweets, and sweetened beverages and juices and lower intakes of red and processed meat than males. The literature exploring the effect of the Mediterranean dietary pattern on CRC in males and females separately [13,14,15,21,22] does not report concordant results, with some studies reporting similar associations for both sexes [15,21], others finding a stronger association among males [13,22], and others among females [14]. We found a stronger effect among males, which could also be explained by differences in the dietary compositions. In our sample, males had higher intakes of fruits and vegetables, fish, and legumes than women. In addition, the longer (for participants highly adherent to the Western dietary pattern) or shorter (for participants highly adherent to the Mediterranean dietary pattern) time that the faecal contents are in contact with the gut mucosa might also be related to CRC risk (the shorter the time, the lower the potential for faecal mutagens to interact with the colon mucosa) [51]. The prevalence of functional constipation is approximately two times higher in women than in men [52], which could be related to the greater effect of the Western pattern on CRC risk in women (accentuation of constipation) and the bigger effect of the Mediterranean pattern among males (increased laxative effect). Other biological mechanisms have been suggested to justify the differential effects of diet in men and women [53], but more research is needed to shed light on this issue. 

The stronger effect of the Western dietary pattern on rectal cancer is also supported by previous research. Most studies that have explored differences by tumour location [9,10,13,14,16,18,19,21,22] have found an effect only for rectal cancer [18] or a stronger effect for rectal than for colon cancer [13,14,16,19,21,22]. Similarly, studies that included the distinction between proximal and distal colon [6,9,10,13,19,21,22] found an effect only for distal cancers [9,10,19,22] or a stronger effect for distal than for proximal cancers [6,21]. This pattern, although not significant, was also observed in our data. As for the distinct effect of the Mediterranean dietary pattern by tumour location, the previous results were mixed, with some studies finding similar associations for all locations [26], others stronger associations for the proximal colon [6], and yet others stronger associations for the distal colon or rectal cancer [13,21,22]. Although there seems to be no clear explanation for the heterogeneous effects of diet on the different tumour subtypes [54], several hypotheses have been proposed. Again, the higher effect of the dietary patterns on distal colon and/or rectal tumours might be related to the time that the faecal content is in contact with the gut mucosa in these localisations compared to the proximal colon. In addition, the proximal and distal colons present molecular differences that could translate into different carcinogenic pathways in these two locations [55]. The greater effects of the Western and Mediterranean dietary patterns found in distal tumours of the colon and/or rectum might be related to the less mature phenotype and lower immune activity of dendritic cells involved in immunologic surveillance at these locations, making them more sensitive to dietary exposures [56]. Additionally, concentrations of the pro-mutagenic lesion O6-methyldeoxyguanosine, a marker of exposure to many NOCs, present in red and processed meat and other processed foods, have been shown to be significantly higher in the distal colon and rectum than in proximal colon tissues [57]. 

Since alcohol is an established risk factor for breast cancer [3], the authors of the EpiGEICAM study did not include it among the food groups to develop the dietary patterns but introduced it as a confounder in the multivariate models. In addition, the WCRF/AICR report [3] considers the evidence of the detrimental effect of alcohol intake on CRC convincing. Therefore, this decision stands valid in the present study. Yet, since moderate alcohol intake has been typically associated with the Mediterranean diet, it could be argued that this difference might affect the comparison of the results between studies. However, most of the previous studies cited excluded alcohol from the PCA analysis and/or included it as a confounder in the regression models [8,9,10,11,12,13,17,18,19,20,21,22,23,25,26]. Those that included alcohol in the PCA analysis found it to be correlated with the Western-type diet [6,16,24] or identified a single alcohol pattern [7,14,15]. 

Among the main limitations of our study was the lack of information on dietary changes during follow-up. The greater consistency and strength of the associations observed when restricting the analyses to the first 10 years of follow-up may indicate that the representativeness of the information collected at the baseline decreased with time, leading to some dilution of the observed associations. However, although less significant, the association of the Western diet with CRC was also considerable for the whole period. In addition, the dietary patterns used were drawn from a dataset of women collected between 2006 and 2011, which can limit their application to the current sample of men and women collected between 1992 and 1996. However, previous research showed that “a posteriori” dietary patterns can be applied to samples different from those from which they were drawn and still be valid [28], even if the patterns are not fully representative of the diet of the population studied. In addition, research that has extracted dietary patterns separately for men and women showed similar results in both groups [10,13,15,58]. Creating exactly the same food groups in the current and the original study was not possible due to differences in the information collected. However, arbitrary decisions in the distribution of foods were avoided by using the MAPAMA data on food consumption in Spain from 1998 [37] and the nutritional information collected in the MCC-Spain study [26]. Finally, the labelling of the patterns was done subjectively by the authors, which could result in two patterns labelled with the same name but representing different dietary habits. To avoid this limitation, we focussed on pattern compositions instead of labelling to compare our results to previous ones. 

The main strength of this study was the considerable size of the EPIC-Spain cohort, which allowed a sufficient number of cases to detect suggestive associations for the overall CRC risk and heterogeneous effects by sex and cancer subtype. Moreover, the longitudinal design of the study prevented reverse causation and avoided a differential recall bias. Furthermore, the dietary patterns not only reflected interactions between foods and nutrients but also represented a great tool for use in nutrition policy, precisely because of the importance of the total diet in health [5]. Finally, only seven cohorts have explored the association between “a posteriori” dietary patterns and CRC risk [8,9,10,12,13,18,24], most of them from Westernised countries with less dietary variability than Mediterranean countries like Spain and, therefore, with a lower capacity to identify associations. The presence of mixed results in the literature clearly signals the need for further studies on the topic to clarify the previous variability in some of the effects found.

## 5. Conclusions

In conclusion, the adoption of dietary habits consistent with the Mediterranean diet, such as a high intake of whole fruits (not juice), vegetables, legumes, whole grains, nuts, vegetable oils, or fish and reduced intake of high-fat dairy products, red and processed meats, refined grains, sweets, caloric drinks, convenience foods, and sauces could decrease the risk of CRC, especially for distal colon and rectal tumours. These results provide new evidence for the role of diet in colorectal cancer risk based on a large prospective cohort from a Mediterranean country.

## Figures and Tables

**Table 1:** Baseline characteristics of the participants of the colorectal cancer sample of the EPIC-Spain study by quartiles of adherence to the Western, Prudent, and Mediterranean dietary patterns.

	Western					Prudent					Mediterranean				
	Q1	Q2	Q3	Q4	*p* ^a^	Q1	Q2	Q3	Q4	*p* ^a^	Q1	Q2	Q3	Q4	*p* ^a^
	*n* = 10,187	*n* = 10,267	*n* = 10,301	*n* = 10,143		*n* = 10,183	*n* = 10,226	*n* = 10,272	*n* = 10,217		*n* = 10,161	*n* = 10,305	*n* = 10,283	*n* = 10,149	
**Alcohol (gr/day)** median (IQR) ^b^	1.60 (0.00; 11.04)	4.75 (0.01; 22.22)	7.36 (0.36; 29.50)	10.28 (1.43; 34.22)	<0.001	3.84 (0.00; 23.77)	6.29 (0.04; 28.46)	5.46 (0.10; 23.22)	5.70 (0.59; 20.90)	<0.001	1.34 (0.00; 10.94)	3.59 (0.00; 17.09)	6.44 (0.49; 25.89)	14.97 (2.63; 40.52)	<0.001
**Energy (Kcal/day)** mean (sd)	1625 (1335; 1988)	1928 (1584; 2335)	2124 (1784; 2536)	2541 (2129; 3027)	<0.001	1830 (1478; 2264)	2092 (1669; 2574)	2067 (1657; 2572)	2185 (1783; 2672)	<0.001	1587 (1297; 1952)	1875 (1579; 2255)	2122 (1797; 2526)	2600 (2222; 3054)	<0.001
**BMI (kg/m^2^)** mean (sd)	28.23 (25.61; 31.33)	27.94 (25.46; 30.85)	27.71 (25.22; 30.41)	27.44 (24.98; 30.15)	<0.001	28.07 (25.46; 30.86)	27.90 (25.37; 30.78)	27.81 (25.30; 30.71)	27.49 (25.09; 30.33)	<0.001	28.07 (25.35; 31.24)	27.80 (25.19; 30.73)	27.67 (25.22; 30.51)	27.77 (25.48; 30.34)	<0.001
**Age at recruitment (years)** median (IQR)	50.89 (44.44; 57.92)	49.28 (43.26; 56.33)	48.25 (42.55; 54.95)	46.31 (41.60; 52.48)	<0.001	48.91 (42.84; 56.69)	48.58 (42.91; 55.54)	48.72 (42.67; 55.58)	48.35 (42.66; 54.93)	<0.001	49.73 (42.97; 57.49)	48.70 (42.52; 55.98)	48.10 (42.54; 54.75)	48.32 (43.11; 54.38)	<0.001
**Colorectal Cancer**					0.140					0.229					0.107
**No**	10,067 (98.82%)	10,120 (98.57%)	10,141 (98.45%)	10,002 (98.61%)		10,021 (98.41%)	10,087 (98.64%)	10,141 (98.72%)	10,081 (98.67%)		10,019 (98.60%)	10,178 (98.77%)	10,148 (98.69%)	9985 (98.38%)	
**Yes**	120 (1.18%)	147 (1.43%)	160 (1.55%)	141 (1.39%)		162 (1.59%)	139 (1.36%)	131 (1.28%)	136 (1.33%)		142 (1.40%)	127 (1.23%)	135 (1.31%)	164 (1.62%)	
**Subtype**					0.535					0.842					0.216
**Proximal**	37 (0.36%)	36 (0.35%)	39 (0.38%)	37 (0.36%)		40 (0.39%)	38 (0.37%)	34 (0.33%)	37 (0.36%)		35 (0.34%)	39 (0.38%)	31 (0.30%)	44 (0.43%)	
**Distal**	43 (0.42%)	54 (0.53%)	62 (0.60%)	46 (0.45%)		58 (0.57%)	49 (0.48%)	49 (0.48%)	49 (0.48%)		55 (0.54%)	44 (0.43%)	54 (0.53%)	52 (0.51%)	
**Rectum**	37 (0.36%)	48 (0.47%)	51 (0.50%)	47 (0.46%)		54 (0.53%)	42 (0.41%)	44 (0.43%)	43 (0.42%)		40 (0.39%)	39 (0.38%)	46 (0.45%)	58 (0.57%)	
**Other colorectal tumours with location non-specified**	3 (0.03%)	9 (0.09%)	8 (0.08%)	11 (0.11%)		10 (0.10%)	10 (0.10%)	4 (0.04%)	7 (0.07%)		12 (0.12%)	5 (0.05%)	4 (0.04%)	10 (0.10%)	
**Sex** *n* (%)					<0.001					<0.001					<0.001
**Male**	2340 (22.97%)	3589 (34.96%)	4318 (41.92%)	5121 (50.49%)		3576 (35.12%)	4203 (41.10%)	3840 (37.38%)	3749 (36.69%)		2243 (22.07%)	3175 (30.81%)	4117 (40.04%)	5833 (57.47%)	
**Female**	7847 (77.03%)	6678 (65.04%)	5983 (58.08%)	5022 (49.51%)		6607 (64.88%)	6023 (58.90%)	6432 (62.62%)	6468 (63.31%)		7918 (77.93%)	7130 (69.19%)	6166 (59.96%)	4316 (42.53%)	
**Physical Activity** *n* (%)					<0.001					<0.001					<0.001
**Inactive**	1048 (10.29%)	1378 (13.42%)	1417 (13.76%)	1561 (15.39%)		1175 (11.54%)	1345 (13.15%)	1383 (13.46%)	1501 (14.69%)		1116 (10.98%)	1260 (12.23%)	1364 (13.26%)	1664 (16.40%)	
**Moderately inactive**	1785 (17.52%)	2117 (20.62%)	2185 (21.21%)	2434 (24.00%)		2064 (20.27%)	2208 (21.59%)	2107 (20.51%)	2142 (20.97%)		1911 (18.81%)	2010 (19.51%)	2108 (20.50%)	2492 (24.55%)	
**Moderately active**	6406 (62.88%)	5902 (57.49%)	5810 (56.40%)	5303 (52.28%)		6117 (60.07%)	5863 (57.33%)	5863 (57.08%)	5578 (54.60%)		6310 (62.10%)	6162 (59.80%)	5871 (57.09%)	5078 (50.03%)	
**Active**	948 (9.31%)	870 (8.47%)	889 (8.63%)	845 (8.33%)		827 (8.12%)	810 (7.92%)	919 (8.95%)	996 (9.75%)		824 (8.11%)	873 (8.47%)	940 (9.14%)	915 (9.02%)	
**Smoking** *n* (%)					<0.001					0.000					<0.001
**Never Smoker**	6609 (64.88%)	5855 (57.03%)	5485 (53.25%)	4764 (46.97%)		5703 (56.01%)	5619 (54.95%)	5707 (55.56%)	5684 (55.63%)	<0.001	6290 (61.90%)	5970 (57.93%)	5587 (54.33%)	4866 (47.95%)	
**Former Smoker**	1699 (16.68%)	1806 (17.59%)	1812 (17.59%)	1905 (18.78%)		1392 (13.67%)	1713 (16.75%)	1936 (18.85%)	2181 (21.35%)		1337 (13.16%)	1643 (15.94%)	1911 (18.58%)	2331 (22.97%)	
**Current Smoker**	1873 (18.39%)	2601 (25.33%)	2998 (29.10%)	3469 (34.20%)		3083 (30.28%)	2888 (28.24%)	2624 (25.55%)	2346 (22.96%)		2530 (24.90%)	2688 (26.08%)	2776 (27.00%)	2947 (29.04%)	
**Unknown**	6 (0.06%)	5 (0.05%)	6 (0.06%)	5 (0.05%)		5 (0.05%)	6 (0.06%)	5 (0.05%)	6 (0.06%)		4 (0.04%)	4 (0.04%)	9 (0.09%)	5 (0.05%)	
**Education** *n* (%)					<0.001					<0.001					<0.001
**No formal Education**	4028 (39.54%)	3613 (35.19%)	3404 (33.05%)	3026 (29.83%)		3985 (39.13%)	3528 (34.50%)	3446 (33.55%)	3112 (30.46%)		4060 (39.96%)	3714 (36.04%)	3339 (32.47%)	2958 (29.15%)	
**Primary School**	3727 (36.59%)	3898 (37.97%)	4042 (39.24%)	4155 (40.96%)		4054 (39.81%)	4136 (40.45%)	3986 (38.80%)	3646 (35.69%)		3838 (37.77%)	3829 (37.16%)	4126 (40.12%)	4029 (39.70%)	
**Secondary/Technical School**	1267 (12.44%)	1530 (14.90%)	1553 (15.08%)	1658 (16.35%)		1290 (12.67%)	1447 (14.15%)	1531 (14.90%)	1740 (17.03%)		1222 (12.03%)	1540 (14.94%)	1523 (14.81%)	1723 (16.98%)	
**University or more**	1101 (10.81%)	1157 (11.27%)	1230 (11.94%)	1232 (12.15%)		780 (7.66%)	1037 (10.14%)	1234 (12.01%)	1669 (16.34%)		984 (9.68%)	1157 (11.23%)	1215 (11.82%)	1364 (13.44%)	
**Unknown**	64 (0.63%)	69 (0.67%)	72 (0.70%)	72 (0.71%)		74 (0.73%)	78 (0.76%)	75 (0.73%)	50 (0.49%)		57 (0.56%)	65 (0.63%)	80 (0.78%)	75 (0.74%)	
**Previous history of CRC** *n* (%)					0.131					0.003					0.867
**No**	9460 (92.86%)	9584 (93.35%)	9641 (93.59%)	9500 (93.66%)		9580 (94.08%)	9559 (93.48%)	9550 (92.97%)	9496 (92.94%)		9504 (93.53%)	9619 (93.34%)	9611 (93.46%)	9451 (93.12%)	
**Mother/Father**	565 (5.55%)	537 (5.23%)	528 (5.13%)	499 (4.92%)		458 (4.50%)	522 (5.10%)	564 (5.49%)	585 (5.73%)		514 (5.06%)	543 (5.27%)	521 (5.07%)	551 (5.43%)	
**Siblings**	122 (1.20%)	111 (1.08%)	92 (0.89%)	97 (0.96%)		98 (0.96%)	109 (1.07%)	116 (1.13%)	99 (0.97%)		100 (0.98%)	104 (1.01%)	111 (1.08%)	107 (1.05%)	
**Unknown**	40 (0.39%)	35 (0.34%)	40 (0.39%)	47 (0.46%)		47 (0.46%)	36 (0.35%)	42 (0.41%)	37 (0.36%)		43 (0.42%)	39 (0.38%)	40 (0.39%)	40 (0.39%)	

^a^ *p*-value calculated ignoring missing values. ^b^ Alcohol was missing for 252 (0.6%) healthy individuals and for 6 (0.01%) colorectal cancer cases.

**Table 2:** Crude and adjusted hazard ratios for the association between colorectal cancer incidence and adherence scores to the Western, Prudent, and Mediterranean dietary patterns for the full period and for the follow-up period 1–10 years.

	Full Follow-Up (>1 year) *n* = 40,898					Full Follow-Up (>1 year) *n* = 40,192					1–10 Years Follow-Up *n* = 40,192				
	Person/Years	Number of Events	HR ^a,b^	(95%CI)		Person/Years	Number of Events	HR ^a,c^	(95%CI)		Person/Years	Number of Events	HR ^a,d^	(95%CI)	
				LL	UL				LL	UL				LL	UL
	**661,313**	**568**				**650,165**	**551**				**351,736**	**211**			
**WESTERN**															
**Quartiles**															
**Q1**	164,274	120	1			161,552	117	1			87,484	45	1		
**Q2**	166,314	147	1.30	1.02	1.66	163,356	145	1.24	0.95	1.60	88,111	56	1.28	0.85	1.91
**Q3**	166,147	160	1.47	1.16	1.87	163,148	151	1.29	0.98	1.71	88,449	53	1.24	0.81	1.89
**Q4**	164,578	141	1.51	1.18	1.93	162,108	138	1.28	0.94	1.73	87,691	57	1.53	0.99	2.36
***p*** **-trend**			0.001					0.143					0.087		
**1SD-increase**			1.15	1.05	1.25			1.06	0.95	1.18			1.17	0.99	1.37
**PRUDENT**															
**Quartiles**															
**Q1**	160,590	162	1			15,7643	158	1			87,125	67	1		
**Q2**	165,016	139	0.84	0.67	1.06	16,2226	132	0.80	0.63	1.01	88,070	40	0.58	0.39	0.86
**Q3**	167,603	131	0.79	0.62	1.00	16,4622	128	0.80	0.63	1.02	88,475	49	0.73	0.50	1.07
**Q4**	168,103	136	0.83	0.65	1.05	16,5675	133	0.87	0.67	1.13	88,066	55	0.85	0.57	1.25
***p*** **-trend**			0.092					0.297					0.547		
**1SD-increase**			0.92	0.85	1.01			0.94	0.85	1.04			0.94	0.81	1.09
**MEDITERRANEAN**															
**Quartiles**															
**Q1**	161,982	142	1			159,681	140	1			87,295	59	1		
**Q2**	166,675	127	0.91	0.71	1.15	164,219	125	0.82	0.64	1.05	88,971	51	0.81	0.55	1.18
**Q3**	167,169	135	0.98	0.77	1.24	164,108	129	0.80	0.62	1.04	88,406	45	0.69	0.46	1.02
**Q4**	165,485	164	1.19	0.94	1.50	162,158	157	0.86	0.66	1.14	87,063	56	0.78	0.52	1.16
***p*** **-trend**			0.107					0.315					0.165		
**1SD-increase**			1.05	0.96	1.14			0.91	0.82	1.01			0.84	0.73	0.98

^a^ Proportional hazards assumption was fulfilled in all cases. ^b^ HR of colorectal cancer stratified by centre and age in 5-year periods. For the Western dietary pattern, also adjusted for adherence to the Prudent and Mediterranean dietary patterns. For the Prudent and Mediterranean dietary patterns, also adjusted for adherence to the Western dietary pattern. ^c^ HR of colorectal cancer stratified by centre and age in 5-year periods adjusted for lifetime alcohol intake, smoking habit, energy intake, BMI, physical activity, education, sex, and family history of colorectal cancer. For the Western dietary pattern, also adjusted for adherence to the Prudent and Mediterranean dietary patterns. For the Prudent and Mediterranean dietary patterns, also adjusted for adherence to the Western dietary pattern. ^d^ HR of colorectal cancer stratified by centre and age in 5-year periods adjusted for lifetime alcohol intake, smoking habit, energy intake, BMI, physical activity, education, sex, and family history of colorectal cancer and including an interaction with time (1–10; >10 years). For the Western dietary pattern, also adjusted for adherence to the Prudent and Mediterranean dietary patterns. For the Prudent and Mediterranean dietary patterns, also adjusted for adherence to the Western dietary pattern.

**Table 3:** Hazard ratios for the association between colorectal cancer incidence and adherence scores to the Western, Prudent, and Mediterranean dietary patterns by sex for the full period and for the follow-up period 1–10 years.

	Full Follow-Up (>1 year)											1–10 Years Follow-Up										
	Male *n* = 15,096					Female *n* = 25,096						Male *n* = 15,096					Female *n* = 25,096					
	Person/Years	Number of Events	HR ^a,b^	(95%CI)		Person/Years	Number of Events	HR ^a,b^	(95%CI)		p-int (Sex)	Person/Years	Number of Events	HR ^a,c^	(95%CI)		Person/Years	Number of Events	HR ^a,c^	(95%CI)		p-int (Sex)
				LL	UL				LL	UL					LL	UL				LL	UL	
	**238,874**	**324**				**411,291**	**227**					**130,848**	**109**				**220,888**	**102**				
**WESTERN**																						
**Quartiles**											0.177											0.005
**Q1**	35,894	56	1			125,658	61	1				19,721	21	1			67,763	24	1			
**Q2**	55,480	72	0.94	0.66	1.35	107,877	73	1.59	1.12	2.26		30,332	22	0.80	0.43	1.46	57,780	34	1.98	1.16	3.36	
**Q3**	67,137	100	1.13	0.79	1.61	96,012	51	1.39	0.94	2.07		36,833	29	0.94	0.53	1.68	51,615	24	1.78	0.99	3.20	
**Q4**	80,364	96	1.05	0.73	1.53	81,744	42	1.55	1.01	2.38		43,961	37	1.27	0.72	2.24	43,730	20	2.16	1.16	4.02	
***p*** **-trend**			0.571					0.068						0.262					0.021			
**1SD-increase**			1.01	0.88	1.15			1.12	0.97	1.30	0.227			1.15	0.92	1.43			1.31	1.06	1.61	0.001
**PRUDENT**																						
**Quartiles**											0.666											0.080
**Q1**	53,559	84	1			104,084	74	1				30,208	32	1.00			56,917	35	1			
**Q2**	65,259	84	0.86	0.63	1.17	96,967	48	0.72	0.50	1.04		35,879	21	0.58	0.34	1.01	52,191	19	0.61	0.35	1.07	
**Q3**	60,548	76	0.86	0.63	1.18	104,074	52	0.73	0.51	1.05		32,837	25	0.78	0.46	1.32	55,638	24	0.70	0.41	1.19	
**Q4**	59,508	80	0.99	0.71	1.37	106,166	53	0.73	0.50	1.07		31,924	31	1.03	0.62	1.73	56,142	24	0.68	0.39	1.18	
***p*** **-trend**			0.922					0.108						0.711					0.200			
**1SD-increase**			0.97	0.86	1.10			0.89	0.78	1.03	0.328			1.01	0.82	1.24			0.88	0.72	1.07	0.007
**MEDITERRANEAN**																						
**Quartiles**											0.505											0.013
**Q1**	33,594	53	1			126,087	87	1				18,897	24	1			68,398	35	1			
**Q2**	49,202	72	0.93	0.65	1.33	115,017	53	0.72	0.51	1.02		27,113	23	0.68	0.38	1.21	61,858	28	0.96	0.58	1.59	
**Q3**	64,411	76	0.77	0.54	1.11	99,697	53	0.88	0.62	1.25		35,111	23	0.55	0.31	0.98	53,296	22	0.95	0.55	1.63	
**Q4**	91,667	123	0.90	0.63	1.28	70,491	34	0.83	0.54	1.27		49,727	39	0.70	0.41	1.19	37,336	17	1.13	0.62	2.08	
***p*** **-trend**			0.486					0.404						0.230					0.811			
**1SD-increase**			0.91	0.80	1.03			0.91	0.79	1.05	0.952			0.80	0.65	0.98			0.99	0.80	1.22	0.006

^a^ Proportional hazards assumption was fulfilled in all cases ^b^ HR of colorectal cancer stratified by centre and age in 5-year periods adjusted for lifetime alcohol intake, smoking habit, energy intake, BMI, physical activity, education, family history of colorectal cancer, and including and interaction with sex. For Western dietary pattern, also adjusted by the adherence to the Prudent and Mediterranean dietary patterns. For Prudent and Mediterranean dietary patterns, also adjusted by the adherence to the Western dietary pattern. ^c^ HR of colorectal cancer stratified by centre and age in 5-year periods adjusted by lifetime alcohol intake, smoking habit, energy intake, BMI, physical activity, education, family history of colorectal cancer and including a three-way interaction with sex and time (1–10; >10 years). For Western dietary pattern, also adjusted by the adherence to the Prudent and Mediterranean dietary patterns. For Prudent and Mediterranean dietary patterns, also adjusted by the adherence to the Western dietary pattern.

**Table 4:** Adjusted hazard ratios for the association between colorectal cancer incidence and adherence scores to the Western, Prudent, and Mediterranean dietary patterns by tumour location for the full period and for the follow-up period 1–10 years.

	Full Follow-Up (>1 year) ^a^															1–10 Years Follow-Up ^a^														
	Proximal					Distal					Rectum					Proximal					Distal					Rectum				
	*n* = 39,784					*n* = 39,839					*n* = 39,821					*n* = 39,784					*n* = 39,839					*n* = 39,821				
	Person/Years	Number of Events	HR ^b,c^	(95%CI)		Person/Years	Number of Events	HR ^b,c^	(95%CI)		Person/Years	Number of Events	HR ^b,c^	(95%CI)		Person/Years	Number of Events	HR ^b,d^	(95%CI)		Person/Years	Number of Events	HR ^b,d^	(95%CI)		Person/Years	Number of Events	HR ^b,d^	(95%CI)	
				LL	UL				LL	UL				LL	UL				LL	UL				LL	UL				LL	UL
	**646,116**	**143**				**646,676**	**198**				**646,429**	**180**				**348,663**	**53**				**349,070**	**73**				**348,905**	**69**			
**WESTERN**																														
**Quartiles**																														
**Q1**	160,768	36	1			160,807	41	1			160,771	37	1			86,874	16	1			86,902	16	1			86,892	12	1		
**Q2**	162,315	35	0.99	0.60	1.62	162,455	53	1.25	0.81	1.93	162,449	48	1.31	0.83	2.07	87,329	12	0.77	0.36	1.67	87,432	22	1.57	0.81	3.05	87,407	18	1.61	0.76	3.41
**Q3**	161,907	35	1.01	0.59	1.74	162,205	60	1.39	0.88	2.20	162,062	48	1.29	0.79	2.12	87,532	14	1.02	0.47	2.22	87,749	19	1.44	0.70	2.93	87,641	16	1.46	0.66	3.25
**Q4**	161,126	37	1.17	0.65	2.08	161,208	44	1.03	0.61	1.73	161,148	47	1.40	0.82	2.39	86,928	11	1.00	0.42	2.35	86,988	16	1.46	0.68	3.15	86,966	23	2.41	1.10	5.30
***p*** **-trend**			0.595					0.897					0.277					0.858					0.439					0.046		
**1SD-increase**			1.02	0.82	1.28			1.03	0.86	1.24			1.05	0.86	1.28			1.02	0.74	1.40			1.17	0.9	1.53			1.38	1.03	1.84
**PRUDENT**																														
**Quartiles**																														
**Q1**	156,521	39	1			156,643	56	1			156,627	53	1			86,242	14	1			86,364	27	1			86,330	21	1		
**Q2**	161,139	35	0.85	0.53	1.35	161,339	47	0.79	0.53	1.17	161,241	41	0.74	0.49	1.12	87,296	13	0.87	0.40	1.87	87,407	11	0.42	0.21	0.85	87,349	13	0.6	0.30	1.21
**Q3**	163,685	32	0.77	0.47	1.26	163,843	48	0.82	0.55	1.23	163,754	44	0.87	0.57	1.33	87,748	11	0.68	0.30	1.55	87,881	16	0.63	0.33	1.19	87,823	19	0.97	0.51	1.84
**Q4**	164,771	37	0.88	0.52	1.48	164,851	47	0.82	0.53	1.28	164,807	42	0.91	0.58	1.45	87,376	15	0.84	0.38	1.86	87,418	19	0.78	0.41	1.49	87,404	16	0.86	0.42	1.73
***p*** **-trend**			0.562					0.429					0.821					0.583					0.529					0.943		
**1SD-increase**			1.02	0.84	1.24			0.89	0.75	1.05			0.98	0.82	1.17			1.01	0.75	1.36			0.89	0.69	1.15			0.98	0.75	1.27
**MEDITERRANEAN**																														
**Quartiles**																														
**Q1**	158,715	34	1			158,892	54	1			158,742	40	1			86,544	15	1			86,674	22	1			86,563	15	1		
**Q2**	163,388	38	1.03	0.64	1.66	163,387	43	0.72	0.48	1.08	163,343	39	0.91	0.58	1.42	88,331	11	0.69	0.32	1.53	88,338	20	0.93	0.50	1.72	88,308	18	1.16	0.58	2.33
**Q3**	163,069	30	0.79	0.47	1.33	163,273	51	0.78	0.52	1.18	163,253	45	1.00	0.63	1.57	87,627	9	0.56	0.24	1.31	87,795	20	0.96	0.51	1.81	87,762	14	0.88	0.41	1.88
**Q4**	160,944	41	0.99	0.58	1.72	161,124	50	0.66	0.41	1.04	161,090	56	1.13	0.69	1.84	86,161	18	1.15	0.53	2.48	86,264	11	0.51	0.23	1.13	86,273	22	1.31	0.63	2.74
***p*** **-trend**			0.744					0.121					0.543					0.805					0.157					0.62		
**1SD-increase**			0.99	0.81	1.22			0.86	0.73	1.02			0.96	0.80	1.15			1.05	0.76	1.44			0.81	0.63	1.03			0.98	0.75	1.29

^a^ The number of tumours with overlapping lesions of colon or those with non-specified locations was 30 in the full follow-up (>1 year) period and 16 in the 1–10 years follow-up period. These cases were excluded from the analyses for the tumour location. ^b^ A proportional hazards assumption was fulfilled in all cases ^c^ HR of colorectal cancer by location stratified by centre, sex, and age in 5-year periods adjusted by lifetime alcohol intake, smoking habit, energy intake, BMI, physical activity, education, sex, and family history of colorectal cancer. For Western dietary pattern, also adjusted by the adherence to the Prudent and Mediterranean dietary patterns. For Prudent and Mediterranean dietary patterns, also adjusted by the adherence to the Western dietary pattern. ^d^ HR of colorectal cancer by location stratified by centre, sex, and age in 5-year periods adjusted by lifetime alcohol intake, smoking habit, energy intake, BMI, physical activity, education, sex, and family history of colorectal cancer and including an interaction with time (1–10; >10 years). For Western dietary pattern, also adjusted by the adherence to the Prudent and Mediterranean dietary patterns. For Prudent and Mediterranean dietary patterns, also adjusted by the adherence to the Western dietary pattern.

## Data Availability

The data of this study is preserved by the EPIC-Spain research group. Data are subject to data sharing agreements and are not publicly available.

## Supplementary Materials

The following supporting information can be downloaded at https://www.mdpi.com/article/10.3390/nu14153085/s1: Table S1. Composition of food groups based on the dietary history questionnaire of the EPIC-Spain study and component loadings obtained with the data of EPIGEICAM study; Figure S1. Adjusted cumulative incidence for colorectal cancer by quartiles of adherence to the Western, Prudent and Mediterranean dietary patterns and sex; Figure S2. Adjusted cumulative incidence for proximal colon, distal colon and rectal cancer by quartiles of adherence to the Western, Prudent and Mediterranean dietary patterns; Figure S3. Non-linear association between colorectal cancer incidence and scores of adherence to Western, Prudent and Mediterranean dietary patterns for the follow-up period 1–10 years. By tumour location.

## References

[1] Sung H., Ferlay J., Siegel R.L., Laversanne M., Soerjomataram I., Jemal A., Bray F. (2021). Global Cancer Statistics 2020: GLOBOCAN Estimates of Incidence and Mortality Worldwide for 36 Cancers in 185 Countries. CA Cancer J. Clin..

[2] Rawla P., Sunkara T., Barsouk A. (2019). Epidemiology of colorectal cancer: Incidence, mortality, survival, and risk factors. Prz. Gastroenterol..

[3] World Cancer Research Fund/American Institute for Cancer Research Continuous Update Project Expert Report 2018. Diet, Nutrition, Physical Activity and Colorrectal Cancer. dietandcancerreport.org.

[4] Marley A.R., Nan H. (2016). Epidemiology of colorectal cancer. Int. J. Mol. Epidemiol. Genet..

[5] Tucker K.L. (2010). Dietary patterns, approaches, and multicultural perspective. Appl. Physiol. Nutr. Metab..

[6] Slattery M.L., Boucher K., Caan B., Potter J., Ma K.-N. (1998). Eating Patterns and Risk of Colon Cancer. Am. J. Epidemiol..

[7] Terry P., Hu F.B., Hansen H., Wolk A. (2001). Prospective study of major dietary patterns and colorectal cancer risk in women. Am. J. Epidemiol..

[8] Fung T., Hu F.B., Fuchs C., Giovannucci E., Hunter D.J., Stampfer M.J., Colditz G.A., Willett W.C. (2003). Major dietary patterns and the risk of colorectal cancer in women. Arch. Intern. Med..

[9] Wu K., Hu F.B., Fuchs C., Rimm E.B., Willett W.C., Giovannucci E. (2004). Dietary patterns and risk of colon cancer and adenoma in a cohort of men (United States). Cancer Causes Control.

[10] Kim M.K., Sasaki S., Otani T., Tsugane S., the Japan Public Health Center-based Prospective Study Group (2005). Dietary patterns and subsequent colorectal cancer risk by subsite: A prospective cohort study. Int. J. Cancer.

[11] Kesse E., Clavel-Chapelon F., Boutron-Ruault M.-C. (2006). Dietary Patterns and Risk of Colorectal Tumors: A Cohort of French Women of the National Education System (E3N). Am. J. Epidemiol..

[12] Butler L.M., Wang R., Koh W.-P., Yu M.C. (2008). Prospective study of dietary patterns and colorectal cancer among Singapore Chinese. Br. J. Cancer.

[13] Flood A., Rastogi T., Wirfält E., Mitrou P.N., Reedy J., Subar A.F., Kipnis V., Mouw T., Hollenbeck A.R., Leitzmann M. (2008). Dietary patterns as identified by factor analysis and colorectal cancer among middle-aged Americans. Am. J. Clin. Nutr..

[14] De Stefani E., Deneo-Pellegrini H., Boffetta P., Ronco A.L., Aune D., Acosta G., Mendilaharsu M., Brennan P., Ferro G. (2009). Dietary patterns and risk of cancer: A factor analysis in Uruguay. Int. J. Cancer.

[15] Miller P.E., Lazarus P., Lesko S.M., Muscat J.E., Harper G., Cross A.J., Sinha R., Ryczak K., Escobar G., Mauger D.T. (2010). Diet Index-Based and Empirically Derived Dietary Patterns Are Associated with Colorectal Cancer Risk. J. Nutr..

[16] Magalhães B., Bastos J., Lunet N. (2011). Dietary patterns and colorectal cancer: A case-control study from Portugal. Eur. J. Cancer Prev..

[17] Safari A., Shariff Z.M., Kandiah M., Rashidkhani B., Fereidooni F. (2013). Dietary patterns and risk of colorectal cancer in Tehran Province: A case–control study. BMC Public Health.

[18] Nimptsch K., Malik V.S., Fung T.T., Pischon T., Hu F.B., Willett W.C., Fuchs C.S., Ogino S., Chan A.T., Giovannucci E. (2014). Dietary patterns during high school and risk of col-orectal adenoma in a cohort of middle-aged women. Int. J. Cancer.

[19] Chen Z., Wang P.P., Woodrow J., Zhu Y., Roebothan B., McLaughlin J.R., Parfrey P.S. (2015). Dietary patterns and colorectal cancer: Results from a Canadian population-based study. Nutr. J..

[20] Azizi H., Asadollahi K., Esmaeili E.D., Mirzapoor M. (2015). Iranian Dietary Patterns and Risk of Colorectal Cancer. Health Promot. Perspect..

[21] Park Y., Lee J., Oh J.H., Shin A., Kim J. (2016). Dietary patterns and colorectal cancer risk in a Korean population. Medicine.

[22] Mehta R.S., Song M., Nishihara R., Drew D.A., Wu K., Qian Z.R., Fung T.T., Hamada T., Masugi Y., da Silva A. (2017). Dietary Patterns and Risk of Colorectal Cancer: Analysis by Tumor Location and Molecular Subtypes. Gastroenterology.

[23] Tayyem R.F., Bawadi H.A., Shehadah I., Agraib L.M., Abu-Mweis S.S., Al-Jaberi T., Al-Nusairr M., Bani-Hani K.E., Heath D.D. (2016). Dietary patterns and colorectal cancer. Clin. Nutr..

[24] Shin S., Saito E., Sawada N., Ishihara J., Takachi R., Nanri A., Shimazu T., Yamaji T., Iwasaki M., Sasazuki S. (2018). Dietary patterns and colorectal cancer risk in middle-aged adults: A large population-based prospective cohort study. Clin. Nutr..

[25] Bahrami A., Houshyari M., Jafari S., Rafiei P., Mazandaranian M., Hekmatdoost A., Sadeghi A., Hejazi E. (2019). Dietary patterns and the risk of colorectal cancer and adenoma: A case control study in Iran. Gastroenterol. Hepatol. Bed Bench.

[26] Castelló A., Amiano P., de Larrea N.F., Martín V., Alonso M.H., Castaño-Vinyals G., Pérez-Gómez B., Olmedo-Requena R., Guevara M., Fernandez-Tardon G. (2018). Low adherence to the western and high adherence to the mediterranean dietary patterns could prevent colorectal cancer. Eur. J. Nutr..

[27] Castelló A., Pollán M., Buijsse B., Ruiz A., Casas A.M., Baena-Cañada J.M., Lope V., Antolín S., Ramos M., Munoz M. (2014). Spanish Mediterranean diet and other dietary patterns and breast cancer risk: Case-control EpiGEICAM study. Br. J. Cancer.

[28] Castelló A., Buijsse B., Martín M., Ruiz A., Casas A.M., Baena-Cañada J.M., Pastor-Barriuso R., Antolín S., Ramos M., Muñoz M. (2016). Evaluating the Applicability of Data-Driven Dietary Patterns to Independent Samples with a Focus on Measurement Tools for Pattern Similarity. J. Acad. Nutr. Diet..

[29] Castelló A., Boldo E., Pérez-Gómez B., Lope V., Altzibar J.M., Martín V., Castaño-Vinyals G., Dierssen-Sotos T., Tardón A., Moreno V. (2017). Adherence to the Western, Prudent and Medi-terranean dietary patterns and breast cancer risk: MCC-Spain study. Maturitas.

[30] Castelló A., de Larrea N.F., Martín V., Dávila-Batista V., Boldo E., Guevara M., Moreno V., Castaño-Vinyals G., Gómez-Acebo I., Fernández-Tardón G. (2017). High adherence to the Western, Prudent, and Mediterranean dietary patterns and risk of gastric adenocarcinoma: MCC-Spain study. Gastric Cancer.

[31] Solans M., Castelló A., Benavente Y., Marcos-Gragera R., Amiano P., Gracia-Lavedan E., Costas L., Robles C., Gonzalez-Barca E., de la Banda E. (2018). Adherence to the Western, Prudent, and Mediterranean dietary patterns and chronic lymphocytic leukemia in the MCC-Spain study. Haematologica.

[32] Riboli E., Hunt K.J., Slimani N., Ferrari P., Norat T., Fahey M., Charrondière U.R., Hémon B., Casagrande C., Vignat J. (2002). European Prospective Investigation into Cancer and Nutrition (EPIC): Study populations and data collection. Public Health Nutr..

[33] Riboli E., Kaaks R. (1997). The EPIC Project: Rationale and study design. European Prospective Investigation into Cancer and Nu-trition. Int. J. Epidemiol..

[34] González C.A., Navarro C., Martínez C., Quirós J.R., Dorronsoro M., Barricarte A., Tormo M.J., Agudo A., Chirlaque M.D., Amiano P. (2004). The European prospective investi-gation about cancer and nutrition (EPIC). Rev. Esp. Salud Publica.

[35] (1997). Relative validity and reproducibility of a diet history questionnaire in Spain. I. Foods. EPIC Group of Spain. European Prospective Investigation into Cancer and Nutrition. Int. J. Epidemiol..

[36] (1997). Relative validity and reproducibility of a diet history questionnaire in Spain. II. Nutrients. EPIC Group of Spain. European Prospective Investigation into Cancer and Nutrition. Int. J. Epidemiol..

[37] Panel de Consumo Alimentario. Ministerio de Agricultura, Pesca y Alimentación. https://www.mapa.gob.es/es/alimentacion/temas/consumo-tendencias/panel-de-consumo-alimentario/.

[38] Castaño-Vinyals G., Aragonés N., Pérez-Gómez B., Martín V., Llorca J., Moreno V., Altzibar J.M., Ardanaz E., de Sanjosé S., Jiménez-Moleón J.J. (2015). Population-based multicase-control study in common tumors in Spain (MCC-Spain): Rationale and study design. Gac. Sanit..

[39] Harrel F. (2015). Regression Modeling Strategies: With Applications to Linear Models, Logistic Regression, and Survival Analysis.

[40] Barbaresko J., Koch M., Schulze M.B., Nöthlings U. (2013). Dietary pattern analysis and biomarkers of low-grade inflammation: A systematic literature review. Nutr. Rev..

[41] Thorburn A.N., Macia L., Mackay C.R. (2014). Diet, Metabolites, and “Western-Lifestyle” Inflammatory Diseases. Immunity.

[42] Ashmore J.H., Rogers C.J., Kelleher S.L., Lesko S.M., Hartman T.J. (2015). Dietary Iron and Colorectal Cancer Risk: A Review of Human Population Studies. Crit. Rev. Food Sci. Nutr..

[43] Jeyakumar A., Dissabandara L., Gopalan V. (2016). A critical overview on the biological and molecular features of red and processed meat in colorectal carcinogenesis. J. Gastroenterol..

[44] Santarelli R.L., Pierre F., Corpet D. (2008). Processed Meat and Colorectal Cancer: A Review of Epidemiologic and Experimental Evidence. Nutr. Cancer.

[45] Fang Y.-Z., Yang S., Wu G. (2002). Free radicals, antioxidants, and nutrition. Nutrition.

[46] Giovannucci E., Willett W.C. (1994). Dietary factors and risk of colon cancer. Ann. Med..

[47] Pelucchi C., Bosetti C., Negri E., Lipworth L., La Vecchia C. (2011). Olive oil and cancer risk: An update of epidemiological findings through 2010. Curr. Pharm. Des..

[48] Turnbaugh P.J., Ridaura V.K., Faith J.J., Rey F.E., Knight R., Gordon J.I. (2009). The Effect of Diet on the Human Gut Microbiome: A Metagenomic Analysis in Humanized Gnotobiotic Mice. Sci. Transl. Med..

[49] Schwabe R.F., Jobin C. (2013). The microbiome and cancer. Nat. Rev. Cancer.

[50] Moszak M., Szulińska M., Bogdański P. (2020). You Are What You Eat—The Relationship between Diet, Microbiota, and Metabolic Disorders—A Review. Nutrients.

[51] Gensollen T., Iyer S.S., Kasper D.L., Blumberg R.S. (2016). How colonization by microbiota in early life shapes the immune system. Science.

[52] Barberio B., Judge C., Savarino E.V., Ford A.C. (2021). Global prevalence of functional constipation according to the Rome criteria: A systematic review and meta-analysis. Lancet Gastroenterol. Hepatol..

[53] Marino M., Masella R., Bulzomi P., Campesi I., Malorni W., Franconi F. (2011). Nutrition and human health from a sex–gender perspective. Mol. Asp. Med..

[54] Huyghe J.R., Harrison T.A., Bien S.A., Hampel H., Figueiredo J.C., Schmit S.L., Conti D.V., Chen S., Qu C., Lin Y. (2021). Genetic architectures of proximal and distal colorectal cancer are partly distinct. Gut.

[55] Missiaglia E., Jacobs B., D’Ario G., Di Narzo A., Soneson C., Budinska E., Popovici V., Vecchione L., Gerster S., Yan P. (2014). Distal and proximal colon cancers differ in terms of molecular, pathological, and clinical features. Ann. Oncol..

[56] Bernardo D., Durant L., Mann E.R., Bassity E., Montalvillo E., Man R., Vora R., Reddi D., Bayiroglu F., Fernández-Salazar L. (2015). Chemokine (C-C Motif) Receptor 2 Mediates Dendritic Cell Recruitment to the Human Colon but Is Not Responsible for Differences Observed in Dendritic Cell Subsets, Phenotype, and Function Between the Proximal and Distal Colon. Cell. Mol. Gastroenterol. Hepatol..

[57] Povey A.C., Hall C.N., Badawi A.F., Cooper D.P., O’Connor P.J. (2000). Elevated levels of the pro-carcinogenic adduct, O6-methylguanine, in normal DNA from the cancer prone regions of the large bowel. Gut.

[58] De Stefani E., Deneo-Pellegrini H., Ronco A.L., Correa P., Boffetta P., Aune D., Acosta G., Mendilaharsu M., Luaces M.E., Lando G. (2011). Dietary Patterns and Risk of Colorectal Cancer: A Factor Analysis in Uruguay. Asian Pac. J. Cancer Prev..

