# Supplementary material for: Adherence to the Western, Prudent and Mediterranean Dietary Patterns and Colorectal Cancer Risk: Findings from the Spanish Cohort of the European Prospective Investigation into Cancer and Nutrition (EPIC-Spain)
Source: Nutrients. 2022 Jul 27;14(15):3085. doi: 10.3390/nu14153085 (PMC9370446; doi:10.3390/nu14153085)
Supplement: Supplementary file 1 [file nutrients-14-03085-s001.zip › nutrients-1802411-supplementary.pdf]

SUPPLEMENTARY MATERIAL.

Table S1: Composition of food groups based on the dietary history questionnaire of the EPIC-Spain study and component loadings obtained with the data of EPIGEICAM study [27].

FOOD GROUP	FOOD[a]	West[b]	Prud[b]	Med[b]
HIGH-FAT DAIRY	Whole-fat milk, milk beverages, curd and yogurt; cream desserts; dairy creams; fraction of unknown fat content dairy[c].	0.60	-0.11	0.20
LOW FAT DAIRY	Semi-skimmed and skimmed milk, milk beverages, curd and yogurt; Cottage or fresh white cheese. portion of unknown fat content dairy[c].	-0.49	0.60	-0.01
EGGS	Eggs.	0.19	0.08	0.16
WHITE MEAT	Poultry and game.	0.08	0.17	0.18
RED MEAT	Red meat (Pork, beef, veal, lamb, etc.); liver; entrails; hamburgers: meatballs; grounded meat; non-specified or mixed meat and derivates.	0.27	0.09	0.22
PROCESSED MEAT	Serrano ham and other cold meat; bacon; other processed meats; non-specified or mixed processed meat.	0.36	0.10	0.26
WHITE FISH	Fresh or frozen white fish (hake, sea bass, sea bream); 2/3 of processed white fish; ½ of fish eggs; ½ of fish liver; 1/3 of non classified fish and seafood.	0.01	0.22	0.34
OILY FISH	Fresh frozen or processed blue fish (tuna, swordfish, sardines, anchovies, salmon); 2/3 of processed blue fish; ½ of fish eggs; ½ of fish liver; 1/3 of non classified fish and seafood.	0.05	0.24	0.44
SEAFOOD/SHELLFISH	Crustaceans and molluscs; 1/3 of non classified fish and seafood.	0.17	0.27	0.35
LEAFY VEGETABLES	Spinach, chard, lettuce and other leafy vegetables.	-0.11	0.34	0.40
FRUITING VEGETABLES	Tomato, eggplant, zucchini, cucumber, pepper, artichoke and avocado.	0.00	0.36	0.45
ROOT VEGETABLES	Root vegetables such as carrots, turnips, radishes, etc.	0.05	0.35	0.44
OTHER VEGETABLES	Cooked cabbage, cauliflower or broccoli, onion, green beans, asparagus, mushrooms, corn, garlic, gazpacho, vegetable soup and other vegetables.<br>Cabbage; mushrooms; grain or pod vegetables; onion and garlic; tail or sprout vegetables; mixed salads or vegetables; non-classified vegetables.	-0.04	0.40	0.42
LEGUMES	Peas, lentils, chickpeas, beans and broad beans	0.21	0.15	0.34
POTATOES	Potatoes and other tubers.	0.17	0.25	0.40
FRUITS	All kind of fruits.	-0.07	0.31	0.31
NUTS	Nuts and seeds.	0.18	0.22	0.29
REFINED GRAINS	Flour, flakes, starch, semolina flour; pasta, rice, other unspecified or combined grains; pasta; other grains; cereal-based products such as pasta; 1/3 of unspecified or mixed bread, biscuits, cookies; ½ of unspecified bread; White bread; 1/3 of rusks, unspecified cookies; ½ of biscuits, white cookies; ½ of unspecified or combined dough or pastry; bread and pizza dough; 2/3 of unspecified white bread, biscuits, or mixes.	0.37	0.15	0.23
WHOLE GRAINS	Unspecified or combined cereal products: ½ of unspecified bread; non-white bread; breakfast cereals; 1/3 of unspecified or mixed bread, biscuits, biscuits; 2/3 of non-white bread, biscuit, unspecified or mixed biscuits; 1/3 of rusks, unspecified biscuits; ½ of biscuits, not white cookies.	-0.43	0.47	0.06
OLIVES AND VEGETABLE OIL	Olives; vegetable oils.	0.12	0.19	0.34
OTHER EDIBLE FATS	Margarine; butter; oil of marine origin; other animal edible fats.	0.22	0.02	0.11
SWEETS	1/3 of biscuits, unspecified cookies; ½ of biscuits, white cookies; 1/2 of biscuits, non-white biscuits; ½ of unspecified or combined dough or pastry; cupcakes; 1/3 of unspecified or mixed bread, biscuits, biscuits; 1/3 of white bread, biscotti, cookies not specified or mixed; 1/3 of non-white bread, biscuit, cookies not specified or mixed; chocolate, candy, bar, pasta, confectionery; non-chocolate confectionery, candied fruit; sorbet, water ice; cakes and cookies	0.35	0.18	0.05
SUGARY	Jam, Honey, Sugar and fruit in sugar syrup.	0.24	0.05	0.00
JUICES	Vegetable and fruit juices.	0.25	0.67	-0.39
CALORIC DRINKS	Sugar-sweetened soft drinks; unclassified non-alcoholic drinks.	0.74	0.21	-0.25
CONVENIENCE FOOD AND SAUCES	Crackers and snack cookies; Egg derivates; 1/3·processed white fish; 1/3·processed blue fish; tomato sauces; other/unsorted or mixed sauces; aioli sauces; mayonnaise and other creamy sauces; snacks; non-dairy creams; fried products; ½·condiments and sauces not classified; vegetarian products and dishes; other dietary products.	0.47	0.12	0.24

[a] Log-transformed centred intake in grams.
[b] West=Western; Prud=Prudent; Med=Mediterranean.
[c] For non-cancer cases: Fraction in high-fat dairy=0.65 of Unknown and Fraction in low-fat dairy=0.35 of Unknown
For breast cancer cases: Fraction in high-fat dairy=0.62 of Unknown and Fraction in low-fat dairy=0.38 of Unknown
For cases of other tumours: Fraction in high-fat dairy=0.68 of Unknown and Fraction in low-fat dairy=0.32 of Unknown

Figure S1: Adjusted[a] cumulative incidence for colorectal cancer by quartiles of adherence to the Western, Prudent and Mediterranean dietary patterns and sex.

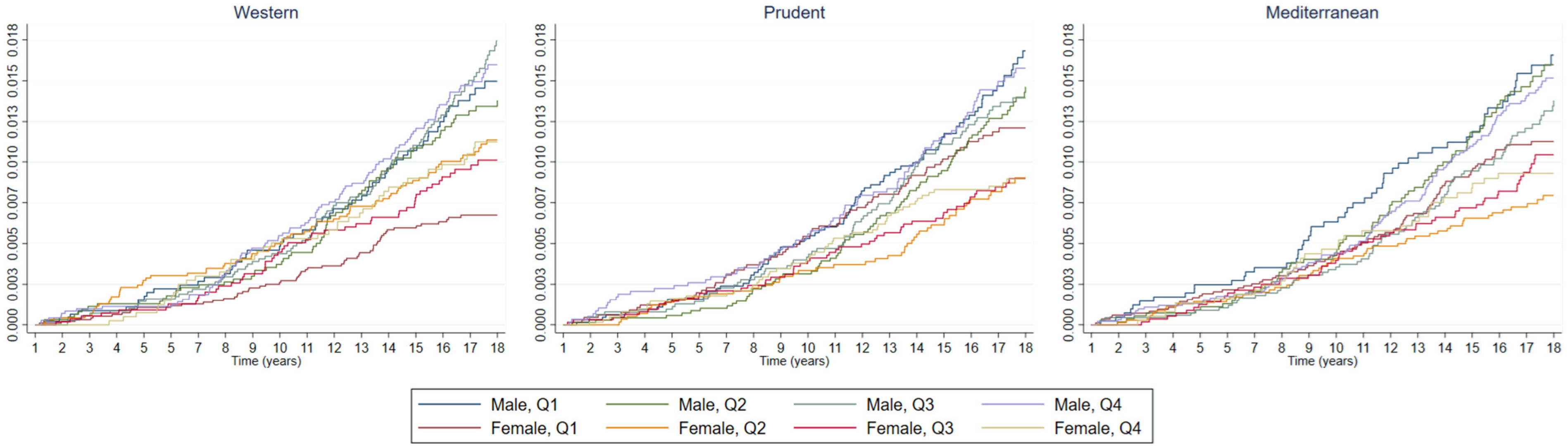

Year	1	2	3	4	5	6	7	8	9	10	11	12	13	14	15	16	17	18
Males at risk	15096	14994	14894	14785	14656	14519	14372	14191	13984	13810	13600	13366	13112	12821	12572	11599	9771	7496
Females at risk	25096	25005	24896	24781	24637	24507	24357	24205	24030	23876	23676	23501	23296	23087	22867	20934	17502	12492

[a] Adjusted by lifetime alcohol intake, smoking habit, energy intake, BMI, physical activity, education, family history of colorectal cancer, age and centre and stratified by sex. For Western dietary pattern, also adjusted by the adherence to the Prudent and Mediterranean dietary patterns. For Prudent and Mediterranean dietary patterns also adjusted by the adherence to the Western dietary pattern.

Figure S2: Adjusted[a] cumulative incidence for proximal colon, distal colon and rectal cancer by quartiles of adherence to the Western, Prudent and Mediterranean dietary patterns.

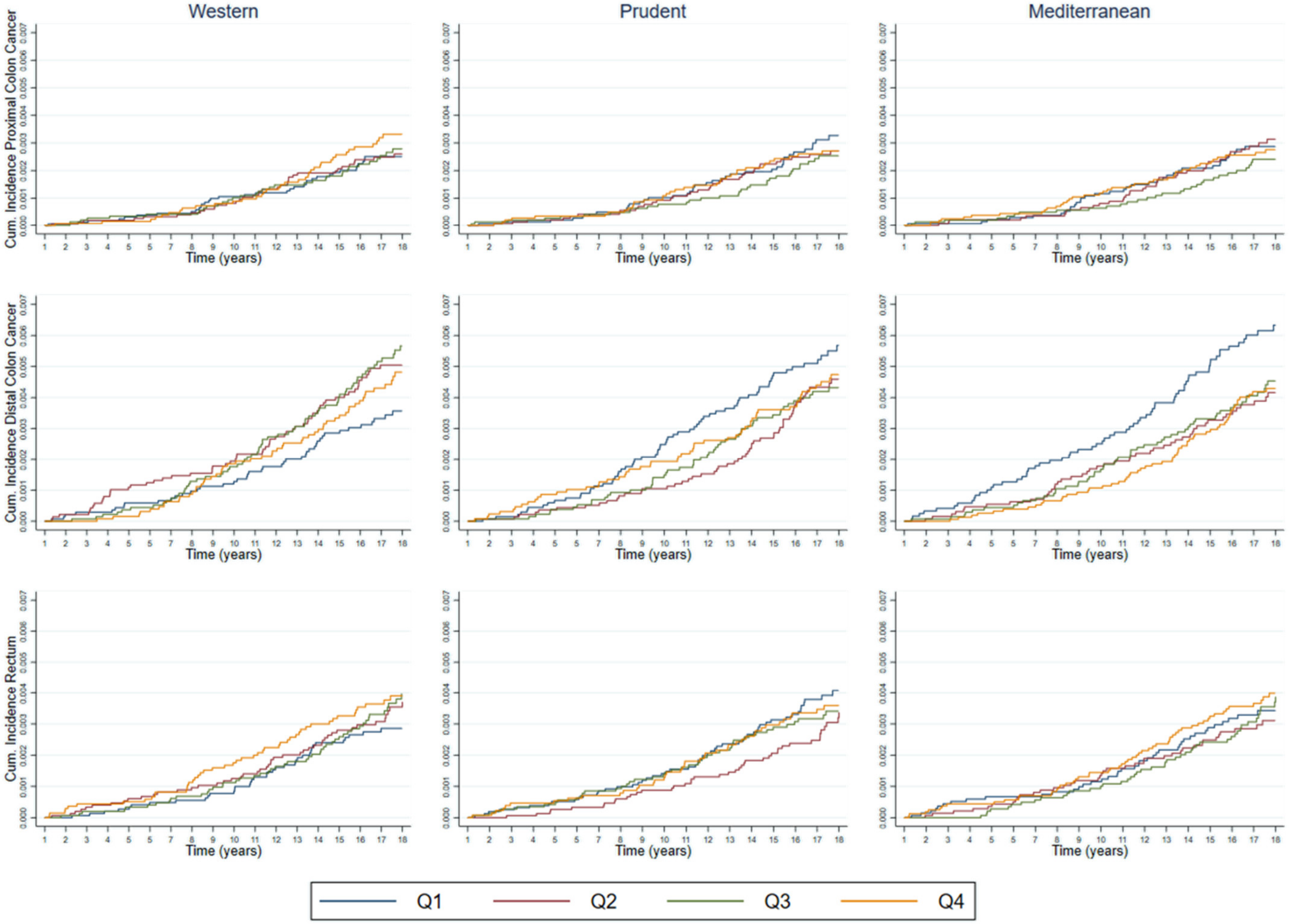

Year	1	2	3	4	5	6	7	8	9	10	11	12	13	14	15	16	17	18
At risk Proximal Colon	40192	39602	39404	39193	38940	38683	38406	38096	37742	37436	37053	36682	36247	35789	35353	32480	27242	19983
At risk Distal Colon	39869	39684	39490	39271	39010	38752	38469	38148	37794	37485	37095	36718	36282	35812	35372	32494	27249	19984
At risk Rectum	39821	39636	39435	39224	38967	38708	38428	38115	37761	37457	37067	36691	36258	35796	35355	32486	27250	19983

[a] Adjusted by lifetime alcohol intake, smoking habit, energy intake, sex, BMI, physical activity, education, family history of colorectal cancer, age and centre. For Western dietary pattern, also adjusted by the adherence to the Prudent and Mediterranean dietary patterns. For Prudent and Mediterranean dietary patterns also adjusted by the adherence to the Western dietary pattern.

Figure S3: Non-linear association between colorectal cancer incidence and scores of adherence to Western, Prudent and Mediterranean dietary patterns for the follow-up period 1-10 years. **By tumour location.**

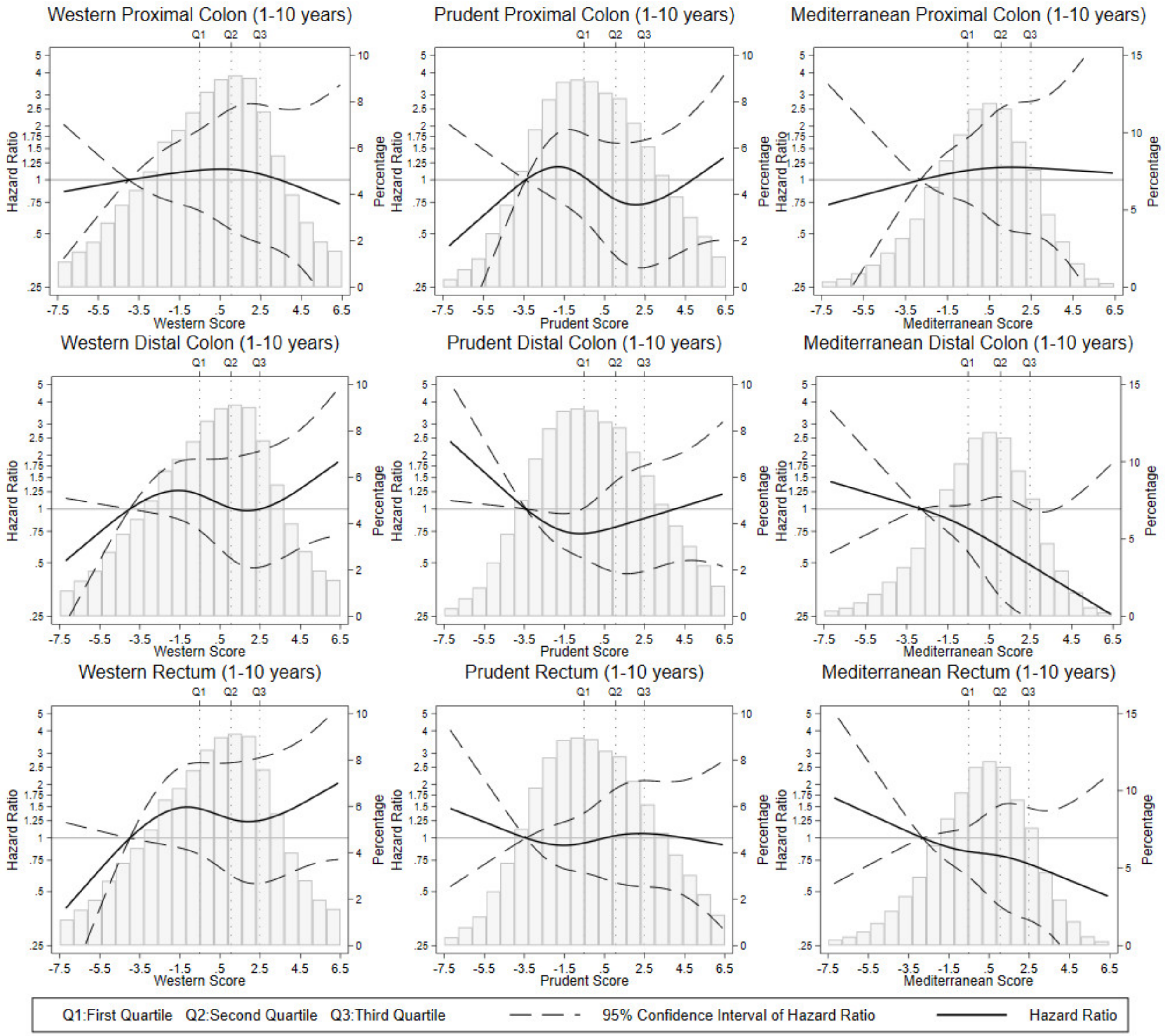

HR of proximal colon, distal colon and rectal cancer stratified by center and age in 5-year periods and adjusted by lifetime alcohol intake, smoking habit, energy intake, BMI, physical activity, education and family history of colorectal cancer. For Western dietary pattern, also adjusted by the adherence to the Prudent and Mediterranean dietary patterns. For Prudent and Mediterranean dietary patterns also adjusted by the adherence to the Western dietary pattern.
